# Editorial: Contemporary Medicine: Making Sense of Implementation Models and Methods

**DOI:** 10.3389/fmed.2022.912045

**Published:** 2022-05-17

**Authors:** Michele M. Ciulla, Ugo Cioffi

**Affiliations:** ^1^Smart Laboratory of Clinical Informatics and Cardiovascular Imaging, CLO, Department of Clinical Sciences and Community Health, University of Milan, Milan, Italy; ^2^Department of Surgery, University of Milan, Milan, Italy

**Keywords:** disease, guidelines, mathematical models, statistic, clinical medicine

## Are Statistical-Mathematical Models Suitable for Studying Human Diseases?

The practice of medicine, defined as “science and art of diagnosing and treating disease or injury” and, more recently, even “promoting *preventive* health care to improve patient well-being,” is more and more guided by statistical-mathematical models that are, indisputably, helpful tools, nonetheless they are subjective, fallible and, thus, confutable. Given that statistics may be more or less adequate, the appropriate use of models and their output can contribute to effective preventive, diagnostic and treatment strategies, however, misuse or misinterpretation of their output can mislead decision-making. In the early'90, to summarize probabilistic data and provide practical guidance to clinical decision making, we sought the birth of the *evidence-based* model and the diffusion of *guidelines* developed accordingly (1, 2). Nowadays this system, subject to public or private funding since guidelines are drafted by invited *experts*, represents the summation of multiple instances, on the one hand the idea of rationalizing the medical intervention, based on available scientific evidences, in order to contain the costs and to build a better system of health care in the public domain and, on the other hand, to provide a legal protection that allows physicians to preserve a wide professional autonomy (Ciulla). Besides, it should also be pointed out that in clinical practice a systematic assessment of health outcomes with post-study probability tests is needed to confirm the intervention effectiveness (3, 4) and, unfortunately, this doesn't happen often.

In this Research Topic, we collected critical reviews and original papers on the use of guidelines in clinical practice to provide and evaluate research methodologies developed for the improvement of patient care, for a better understanding and guiding of clinical decisions, as well as the implementation of new models.

The *promise of digital technologies and data science* to transform healthcare (5) is discussed in the light of compelling ethical, legal, and social challenges, by Cordeiro. When processing large quantities of health data, from different sources, the Author, against the risks of *dehumanization of care*, claims a *normative framework* in order to promote fairness, inclusiveness, creativity and innovation in health.

The goal of *implementation science* is to *close the gap between evidence-based practices and the extent to which research findings are integrated into real world settings and practices* (6). In such perspective, Huybrechts et al. in their review discuss the implementation of *complex interventions within primary care practices* by mapping existing theories, models, and frameworks from implementation science and combined insights across various disciplines. As a part of efforts intended to improve goal oriented care, self-management, and inter-professional collaboration, the Authors summarize three core phases to develop an overarching implementation model, development, translation and sustainment, and three main components, the intended change, the context and the implementation strategies.

A method to *close the gap between research and practice* consists in mixing design components of clinical effectiveness and implementation research in *effectiveness-implementation hybrid design* studies; unfortunately, translating these strategies into routine practice, especially in resource-constrained settings, is an arduous task. The results of a primary care-based integrated mobile health intervention for stroke management in Rural areas of China is discussed in the original paper by Gong et al. to describe the implementation indicators, related enablers and barriers, and illustrate some potential impact pathways that may influence the effectiveness of the intervention. The key factors identified to build an effective *doctor-patient alliance*, based on acceptance and fidelity on voice messages and follow-up visits, consists in supporting village doctors in *clinical decision-making* by training, financial compensation, and support from experienced physicians.

Statistical-mathematical models are often used to predict event outcomes and *nomograms*, representing geometrically the intersection of variables, are practical tools to provide rapid calculations. In the paper by Xu et al., a predictive model, consisting in a nomogram for identifying patients with acute decompensated cirrhosis at high-risk for readmission to the hospital, is proposed; the model, developed retrospectively on four independent clinical and laboratory indicators for each patient, may facilitate the development of effective interventions to reduce readmission rates.

Another predictive model is developed by Yu et al. combining data visualization and *machine learning* to predict the metabolic syndrome phenotype, especially in non-obese subjects where prevention is still challenging. As we know, *artificial intelligence* uses algorithms to learn and improve from experience and/or data without being explicitly programmed giving a perspective on the complicated relationships between metabolic components and potential risk factors.

Fragility fractures, even as pain experience, are very common in the elderly and, often, correlated; in the review by Chen et al. this relationship is analyzed supporting that frail patients with fractures were suffering from a continuous risk of pain exceeding the “typical” length of time assumed as essential for curing and resolution of pain. Thus, medical teams should develop treatment and rehabilitation protocols to prevent or reduce the pain of post-fracture, including meditation, exercises, and integrated physical treatment.

The *zero-markup policy* for essential drugs, a central point of the Chinese health reform started in 2009 (7), plays an important role in decreasing the cost of drugs for chronic diseases, such as type 2 diabetes, hypertension, metabolic syndrome, coronary heart disease, and cancer; Liu et al. in their systematic review discuss the effects of this policy on healthcare costs and utilization in China in the years 2015–2021 supporting a lower drug cost to patients but a rise in other expenditure categories such as healthcare services “induced” by physicians or response to unmet needs in the population.

High-dimensional data are unintuitive and difficult to interpret and derive insights, in the perspective article Bae et al. suggest to dimensionally reduce data by using *pattern identification* found in traditional Asian medicine, a diagnostic system using a limited amount of computation, to evaluate patient's clinical symptoms and signs and classify them. While this approach may appear biased by the underpinned *heuristic system* of belief in comparison to *pathogen-based diagnosis*, it provides an intuitive foundation for *inductive reasoning*.

Finally, when facing *how to build a better healthcare system*, a model of prevention/intervention, with a plausible design, is unavoidable, nonetheless, in the clinical setting, the application of a model requires a verification strategy, with a systematic assessment of its impact on health outcomes with post-study probability testing (Ciulla), and a continuous on-going refinement.

## Author Contributions

Both authors listed have made a substantial, direct, and intellectual contribution to the work and approved it for publication.

## Conflict of Interest

The authors declare that the research was conducted in the absence of any commercial or financial relationships that could be construed as a potential conflict of interest.

## Publisher's Note

All claims expressed in this article are solely those of the authors and do not necessarily represent those of their affiliated organizations, or those of the publisher, the editors and the reviewers. Any product that may be evaluated in this article, or claim that may be made by its manufacturer, is not guaranteed or endorsed by the publisher.
